# Public reporting as a prescriptions quality improvement measure in primary care settings in China: variations in effects associated with diagnoses

**DOI:** 10.1038/srep39361

**Published:** 2016-12-20

**Authors:** Yuqing Tang, Chaojie Liu, Xinping Zhang

**Affiliations:** 1 School of Medicine and Health Management, Tongji Medical College, Huazhong University of Science and Technology, Wuhan, 430030, P.R. China; 2 School of Psychology and Public Health, La Trobe University, Melbourne, Victoria 3086, Australia

## Abstract

The overprovision and irrational use of antibiotics and injections are a major public health concern. Public reporting has been adopted as a strategy to encourage good prescribing practices. This study evaluated the effects of public reporting on antibiotic and injection prescriptions in urban and rural primary care settings in Hubei province, China. A randomized control trial was conducted, with 10 primary care institutions being subject to public reporting and another 10 serving as controls. Prescription indicators were publicly reported monthly over a one-year period. Prescriptions for bronchitis, gastritis and hypertension before and after the intervention were collected. Difference-in-difference tests were performed to estimate the effect size of the intervention on five prescription indicators: percentage of prescriptions containing antibiotics; percentage of prescriptions containing two or more antibiotics; percentage of prescriptions containing injections; percentage of prescriptions containing antibiotic injections; and average prescription cost. Public reporting had varied effects on prescriptions for different diagnoses. It reduced antibiotic prescribing for gastritis. Prescriptions containing injections, especially antibiotic injections, also declined, but only for gastritis. A reduction of prescription costs was noted for bronchitis and gastritis. Public reporting has the potential to encourage good prescribing practices. Its effects vary with different disease conditions.

Antibiotic abuse and the overuse of injections has been identified as a major challenge involved in the irrational prescription of medicines^1^. This has remained common, especially in low and middle income countries^1^,^2^,^3^, and is associated with antimicrobial resistance (AMR), adverse drug events and the transmission of diseases. Loss of productivity, poorer health outcomes and greater health care costs associated with the irrational use of antibiotics and injections have attracted great concern globally^4^. China has perhaps suffered the most. A growing body of literature has revealed a very high level of use of antibiotics and injections in China, especially in primary care institutions^5^,^6^,^7^.

Increased AMR and the spread of blood-borne infections has triggered a surge of research into interventions on prescribing practices^8^,^9^,^10^,^11^. Physicians are therefore targeted for interventions^12^. Well-documented evidence shows that audits and feedback on prescribing performance can result in a small to moderate change in the prescribing practices of physicians (ranging from a 16% decrease to 70% increase in compliance with prescription guidelines)^9^. Although recent studies reported a relatively stronger effect of “audit and feedback” when it was combined with “peer academic detailing”^13^,^14^, the enhanced effect can often be offset by the resources required and practical considerations^15^. A review by the Cochrane Collaboration^9^ concluded that intensive feedback may have a greater potential given that the tested “feedback” interventions are usually confidential and contain only benchmarking on average.

There is growing research interest in the role of public reporting for improving patient care, especially in developed countries^16^,^17^. Public reporting usually involves three broad types of information: health care outcomes; provider performance; and patient experience^16^,^18^,^19^. It is widely believed that public reporting can enable consumers to make better informed choices and improve health care practices. Patients may modify their choice of providers or other decisions on the basis of the available performance measures^20^. Although in some health systems, consumers have limited choice^21^,^22^, health care providers may still face pressure from managerial interventions and social expectations to change their practices^23^.

Extensive studies have been undertaken to evaluate the effectiveness of public reporting on patient care outcomes. Five systematic reviews synthesized the findings of these studies^24^,^25^,^26^,^27^,^28^, but failed to reach a definitive conclusion. The heterogeneity of the outcomes and the moderate strength of the small body of evidence available make it difficult to draw a definitive conclusion^26^,^27^,^28^. The systematic reviews identified a paucity in the literature in relation to the impact of pubic reporting on primary care, outpatient services, and the practices of individual physicians. Reporting of aggregated measures of quality and safety remains relatively uncommon.

This study aimed to determine the impact of public reporting on prescribing practices of physicians in primary care settings in China. Unlike previous efforts made by Yang’s^29^ and Wang’s^30^ teams, where the impact of public reporting on prescriptions was examined through a single disease condition (upper respiratory tract infections) or using a single prescribing indicator (percentage of prescriptions containing injections) over a short period of time (4 months), this study evaluated the impact of public reporting on the use of antibiotics and injections for a range of conditions over a relatively longer period of time (1 year). We chose three conditions for the purpose of this study: bronchitis, gastritis and hypertension. Those are common conditions that present in primary care settings in China^31^,^32^. But the medical treatment regimens for the three conditions vary considerably.

## Results

A total of 36,121 prescriptions (24,580 in the intervention and 11,541 in the control groups) for bronchitis prescribed by 152 physicians (74 in the intervention and 78 in the control groups) were acquired (Table 1). The patients with bronchitis had an average age of 27.79 years, younger than those with gastritis (mean age = 49.72, *p* < 0.001) or hypertension (mean age = 58.29, *p* < 0.001).

For gastritis, 14,849 prescriptions (7,490 in the intervention and 7,359 in the control groups) prescribed by 155 physicians (73 in the intervention and 82 in the control groups) were acquired.

For hypertension, 18,376 prescriptions (10,688 in the intervention and 7,688 in the control groups) prescribed by 161 physicians (78 in the intervention and 83 in the control groups) were acquired.

There were small (but statistically significant) differences between the intervention group and the control group in relation to the characteristics of the prescription recipients (*p* < 0.05), except for “age” in the patients with hypertension recipients (*p* = 0.084). There were no significant differences between the intervention group and the control group in relation to the characteristics of the prescribers except for “professional title” (*p* < 0.05).

Overall, the patients with bronchitis had the highest percentage of prescriptions containing antibiotics (ranging from 77.53% to 95.20%), which was followed by those with gastritis (ranging from 38.22% to 66.61%). A few patients (ranging from 2.42% to 9.95%) with a recorded diagnosis of hypertension were also prescribed with antibiotics.

**Figure 1:**
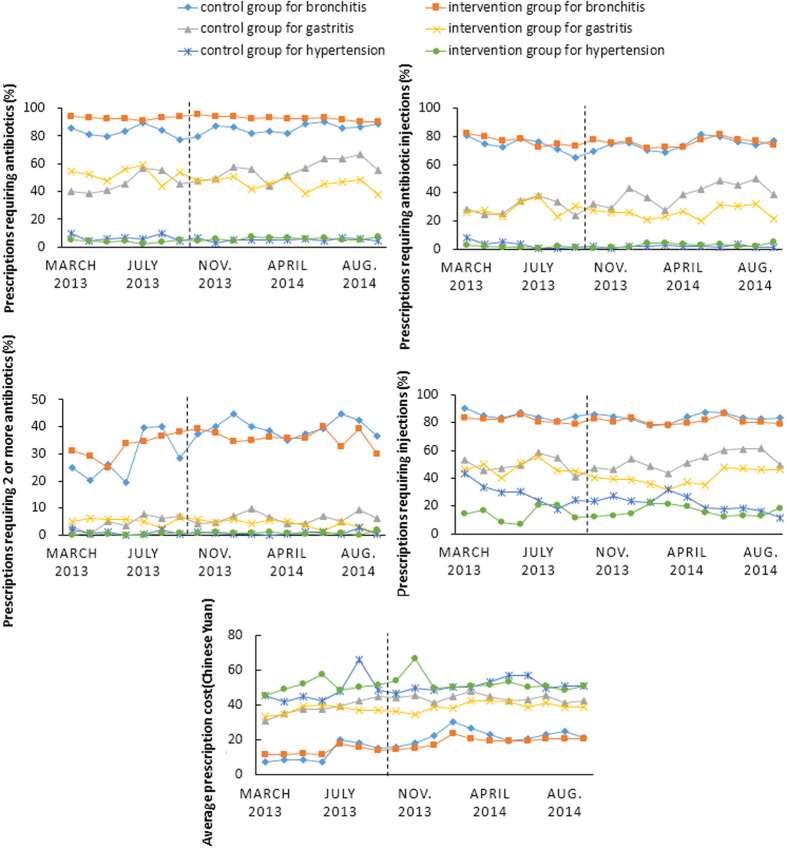
Monthly results of prescribing indicators over time. To make a clearer view, the average cost of bronchitis were 10 minus on every single dot for both intervention and control groups.

Similar patterns of results were found in relation to the use of injections and the combined use of antibiotics and injections (Fig. 1). The percentage of prescriptions containing injections was very high (ranging from 78.46% to 90.44%) for bronchitis, followed by that for gastritis (ranging from 31.82% to 61.31%). About 6.93% to 43.60% of the patients with hypertension were prescribed with injections. For bronchitis, 83.89% of antibiotics were administered through injections, compared with 62.81% for gastritis and 42.73% for hypertension.

The average prescription costs were low. The expenditure on prescriptions for the patients with hypertension was higher (ranging from 42.14 to 66.99 Chinese Yuan) compared with those for bronchitis (ranging from 17.61 to 40.16 Chinese Yuan) and gastritis (ranging from 30.86 to 47.94 Chinese Yuan).

The public reporting intervention resulted in mixed effects on prescribing practices (Table 2). The DID analyses show that the intervention resulted in a decline in the use of antibiotics, with a 12.72% reduction (*p* < 0.001) for gastritis. The intervention also reduced the combined use of antibiotics for bronchitis (3.79% decrease, p = 0.005). The interventional effect of public reporting on the combined use of antibiotics for bronchitis was not detected in the time trend analysis (Supplementary file). The intervention increased the use of antibiotics for hypertension (2% increase, *p* = 0.008), albeit at a low level overall.

The reduction in the use of injections was significant only for the patients with gastritis (10.59% decrease, *p* < 0.001), which included prescriptions requiring antibiotic injections (10.73% decrease, *p* < 0.001). There was a slight increase (2%) in the use of injections for the patients with bronchitis (*p* = 0.012).

The public reporting reduced the costs of prescriptions, but this was only statistically significant for the patients with gastritis (5.71% decrease, *p* = 0.005). and bronchitis (7.90% decrease, *p* < 0.001). The interventional effect of public reporting on the cost of prescriptions for bronchitis was not detected in the time trend analysis (Supplementary file).

## Discussion

This study provides new evidence on the potential of public reporting for encouraging good prescribing practices in primary care settings. Our results suggest that public reporting can reduce antibiotic prescriptions for patients with gastritis. Significant reductions in the percentage of prescriptions requiring injections (including antibiotic injections) are also evident for patients with gastritis.

High levels of injection usage are evident for all of the three disease conditions. Over 80% of the patients with bronchitis and around 50% of the patients with gastritis were prescribed with injections. About 20% of the patients with hypertension were also given injections. The public reporting intervention only had an effect on reducing injections for gastritis. There was a slight increase (2%) in the use of injections for the patients with bronchitis. A lack of proper compensation mechanism is often blamed for the shortage of achievements of China’s National Essential Medicines Program (NEMP)^33^,^34^. When the 15% markup policy for prescribed medicines was abandoned as part of the NEMP, health care providers had to shift their attention to services that could make up the loss (because of the shortage of government funding)^35^. Although there is no profit attached to prescribed medicines, a fee can be charged for administering injections or infusions. This has jeopardized government efforts to reduce the use of injections. Such a situation is further complicated by patient preference. In China, patients often perceive injections as being powerful, fast-acting, and longer lasting than oral pills^36^. This preference may have initially been supplier induced but it now seems self-sustaining. The distorted perception of patients puts additional pressures on prescribers^5^, which can exacerbate the overprovision and irrational use of injections. A qualitative study described how Chinese patients seek medical attention for the common cold^37^. Self-medication is often the first choice. But if symptoms persist after over-the-counter remedies have been taken, the patients are likely to request injectable treatments from a health worker. This is simply because “pills have failed’.

The expectation that public reporting would decrease injection usage may still hold promise. In this study, we observed a decline in injection usage in gastritis patients. Clearly, the dynamics of medical encounters vary for different disease conditions. While patients with bronchitis are more likely to anticipate and request injections, patients with gastrointestinal disorders including gastritis are among those with the highest adherence to medical recommendations^38^. This may explain the differing effects of public reporting on prescribing practices for different disease conditions. Obviously, interventions targeting consumers such as social marketing and health literacy development are equally, if not more important than interventions targeting prescribers.

In this study, we found public reporting had a limited effect on reducing prescription costs. Decreased expenditure on prescriptions was not found for patients with higher bills, such as those with hypertension who have to take lifelong medications. However, expenditure on prescriptions is already quite low in primary care settings thanks to the NEMP. Meanwhile, increased insurance coverage has resulted in lifting the financial burden of patients^39^.

Overall, the antibiotic usage for bronchitis is still too high. Empirical evidence shows that initial antibiotic therapy in bronchitis patients is not necessary unless pneumonia is suspected^40^. But antibiotics are frequently overprescribed for bronchitis even in developed countries^41^,^42^. The level of antibiotic usage for bronchitis unveiled in this study is consistent with findings of previous studies. Two recent studies in China reported 52.3% of antibiotic use for bronchitis in tertiary hospitals^43^ and 93.5% of antibiotic use for bronchitis in primary care facilities^44^. What is even more concerning is that the majority of the prescribed antibiotics for bronchitis were administered through injections, and public reporting failed to reduce the injection rates.

There is paucity in the literature documenting the effect of public reporting on prescribing practices in China. A previous study of patients with upper respiratory tract infections^29^ found a significant reduction in combined use of antibiotics as a result of a four-month reporting intervention; but no changes in injection prescriptions were observed. These results are similar to our findings in this study.

A slight increase in the percentage of prescriptions requiring antibiotics was observed for the patients with hypertension in this study, as was the percentage of prescriptions requiring injections for those with bronchitis. Because only one diagnosis was recorded in each prescription, we are not able to exclude the possibility that antibiotics might have been prescribed for a condition other than hypertension or bronchitis. Further studies are also needed to explore the potential unintended consequences of publicly reporting quality information^45^. The depth of clinical coding is not sufficient to allow us to determine the cause (e.g., bacterial, virus, or other) of bronchitis and gastritis and the phases of hypertension. Ideally, the whole sample of prescriptions should be included in the data analyses with proper risk-adjustments. Unfortunately, we are not able to do so due to the unavailability of relevant data. However, the selection of the three disease conditions in this study provided us with some unique insights into the impact of public reporting interventions.

The public reporting of prescription indicators at physician and facility levels can make a difference to prescribing practices, such as reductions in the use of antibiotics and injections. The effects of public reporting depend on the disease conditions. Prescribers are subject to pressure from policy and organizational environments, as well as consumer expectations. Special consideration should be given to the context in which public reporting interventions are placed.

## Methods

We conducted a randomized controlled trial that was designed to comply with the guidelines of the Consolidated Standards of Reporting Trials (CONSORT).

### Study setting

This study was undertaken in QianJiang city of Hubei province. Hubei is located in central China with a population of over 61 million. The average annual income per capita ranks in the middle range of all provinces: 6,898 Yuan for rural and 18,374 Yuan for urban residents (in 2012). QianJiang has a population of about 950,400 and 47.5% reside in the rural area. In 2012, QianJiang produced a GDP of 49.3 billion (Yuan). The annual average income per capita reached 8785 Yuan for rural and 17451 Yuan for urban residents.

### Randomization

QianJiang has 20 primary care institutions, on average, each institution is 10 kilometers away from the nearest counterpart. All participated in this study. We used matched-pair cluster randomization to assign the participating institutions into the intervention and control groups.

1. A TOPSIS (Technique for Order Preference by Similarity to Ideal Solution) score was calculated for each institution based on six indicators: local population size, number of beds, number of physicians, annual outpatient visits, annual episodes of admissions, and annual revenue from drug sales (Supplementary file).
2. The participating institutions were sorted in an ascending order according to the TOPSIS score, and adjacent institutions were paired.
3. For each pair, we flipped a coin to randomly assign one into the intervention group and another into the control group”.

More details about the research setting, trial design, intervention strategies and the CONSORT checklist can be found in the study protocol published elsewhere^46^. This study obtained approval from the Ethics Review Committee of Tongji Medical College, Huazhong University of Science and Technology (No. IORG0003571). Informed consent was obtained from all participated physicians.

### Interventions

The public reporting contained information about: (1) percentage of prescriptions containing antibiotics; (2) percentage of prescriptions containing injections; and (3) average expenditure per prescription. The indicators were calculated by the research team using the computerized hospital information management systems of the participating institutions. They were ranked in ascending orders at the institution level and the prescriber level within an institution.

The ranking information was disseminated through a poster displayed in the public place of the institutions, handout brochures, and a report submitted to the local health authority. The posters and brochures included a brief introduction about the purpose of the reporting. It was made clear that health risks are associated with excessive use of antibiotics and injections.

On the ninth day of each month during the intervention period, four or five researchers were dispatched to disseminate the reporting information in the intervention sites. To maximize compliance, the local health authority issued a policy to protect the information dissemination activities. Meanwhile, the research team inspected the intervention sites irregularly. Damaged posters, if found, were replaced immediately.

### Data collection

Data used in this study came from two sources. Prescription data were extracted from the electronic medical records, which included the name and work units of the prescribers, the time when the prescriptions were written, the demographic characteristics of the recipients (age, sex and insurance entitlements), the reason for the prescriptions (only one diagnosis was recorded for each prescription), as well as the details of medicines prescribed (drug name, administration route, dosage, frequency, and costs). Data on the characteristics of prescribers were collected through a self-administered questionnaire, which included their name, age, sex, level of education, professional title, and income. The two data sets were linked by matching the names of the prescribers.

The prescriptions with a recorded diagnosis of bronchitis, gastritis or hypertension were identified and collated for data analyses. We chose these three conditions for several reasons. First, risk-adjustment is needed to assess the quality of prescriptions. But unfortunately, a case-mix system for such risk-adjustment in primary care settings is not available yet^47^. Second, prescriptions for the three conditions accounted for a relatively large portion (approximately 20%) of the entire prescriptions collected. Third, theoretically, the use of antibiotics and injections for the three conditions varies considerably, which allows us to capture a good picture of prescribing patterns.

We started pubic reporting interventions in October 2013. Prescriptions one year before and one year after the intervention were collected.

### Statistical analysis

The International Network for Rational Use of Drugs developed a list of prescribing indicators^48^, which have become widely accepted internationally. We selected five indicators for the purpose of this study, which covered the type (antibiotics) and administration route (injections) of medicines that had been most frequently abused and the cost of medicines which has attracted a great deal of concern in China.

1. Percentage of prescriptions containing antibiotics;
2. Percentage of prescriptions containing two or more antibiotics;
3. Percentage of prescriptions containing injections;
4. Percentage of prescriptions containing antibiotic injections;
5. Average prescription cost (Chinese yuan).

We calculated the monthly results of the five indicators in relation to the three disease conditions, and depicted the results on a connected dot-line plot with a view to demonstrate time changes (before and after interventions) and differences between the intervention and control groups.

Differences between the intervention group and the control group in relation to the characteristics of both recipients and prescribers were tested using student t tests for continuous variables and Chi-square tests for categorical variables.

We adopted a difference-in-difference (DID) approach to test the effects of the intervention. Multivariate regression models were established with the five prescription indicators as dependent variables. Logit regression models were applied for the binary dependent variables, such as the usage of antibiotics, injections, and antibiotic injections. We used least squares regression models for the prescription costs (natural logarithm transformed). In the regression models, a dummy variable “intervention” was entered indicating whether a prescription was in the intervention or control group; a dummy variable “pre-post” was entered indicating whether a prescription was written before or after the intervention; and the effects of the intervention were tested through the interaction between the two dummy variables (“intervention”*“pre-post”).

To control confounding factors, patient characteristics (age, sex, and health insurance coverage) and prescriber characteristics (age, sex, level of educational, professional title, and income) were also introduced into the regression models. Three levels of measurements (prescription- prescriber-institutions) were involved in this study. We randomized the study sample at the institution level. The data analyses adjusted clustering at the prescriber level. We used mixed effect models which accounted for random intercepts of individual prescribers. The effect margins were calculated and reported.

For the indicators that showed a time trend, we performed a controlled segmented regression analysis (Supplementary file). But for simplicity, we only presented the findings of DID analyses if no inconsistencies were found between the two methods.

The statistical analyses were performed using Stata 12.1 (Stata Corp).

## Additional Information

**How to cite this article**: Tang, Y. *et al*. Public reporting as a prescriptions quality improvement measure in primary care settings in China: variations in effects associated with diagnoses. *Sci. Rep.* **6**, 39361; doi: 10.1038/srep39361 (2016).

**Publisher's note:** Springer Nature remains neutral with regard to jurisdictional claims in published maps and institutional affiliations.

## Supplementary Material

Supplementary File

## Figures and Tables

**Table 1:** Characteristics of recipients (patients) and prescribers (physicians) of prescriptions.

Prescription	Bronchitis		Gastritis		Hypertension	
	Intervention	Control	Intervention	Control	Intervention	Control
**Characteristics of recipients**						
Age (years, mean ± SD)	25.7 ± 24.9	32.2 ± 25.7	50.5 ± 18.5	49.0 ± 20.0	58.1 ± 15.2	58.5 ± 15.8
Male (n, %)	12269 (49.9)	6044 (52.4)	3189 (42.6)	3330 (45.3)	4932 (46.1)	3756 (48.9)
NCMS coverage (n, %)	21925 (89.2)	10131 (87.8)	6832 (91.2)	6626 (90)	10337 (96.7)	7400 (96.3)
**Characteristics of prescribers**						
Age (years, mean ± SD)	39.05 ± 8.25	43.42 ± 9.31	40.11 ± 8.58	43.41 ± 9.04	39.32 ± 8.30	43.28 ± 8.91
Male (n, %)	55 (74.32)	58 (74.36)	55 (75.34)	62 (75.61)	57 (73.08)	60 (72.29)
**Level of education (n, %)**						
*Vocational*	19 (25.68)	29 (37.18)	19 (26.03)	29 (35.37)	21 (26.92)	31 (37.35)
*Associate degree*	44 (59.46)	37 (47.44)	41 (56.16)	42 (51.22)	44 (56.41)	39 (46.99)
*Tertiary degree*	11 (14.86)	12 (15.38)	13 (17.81)	11 (13.41)	13 (16.67)	13 (15.66)
**Professional title (n, %)**						
*Assistant*	21 (28.38)	17 (21.79)	20 (27.40)	17 (20.73)	26 (33.33)	17 (20.48)
*Resident*	33 (44.59)	14 (17.95)	30 (41.10)	15 (18.29)	29 (37.18)	16 (19.28)
*Attending*	18 (24.32)	42 (53.85)	20 (27.40)	46 (56.10)	19 (24.36)	44 (53.01)
*Chief*	2 (2.70)	5 (6.41)	3 (4.11)	4 (4.88)	4 (5.13)	6 (7.23)
**Income (Chinese Yuan)**						
* < 1500*	17 (22.97)	21 (26.92)	16 (21.92)	20 (24.39)	15 (19.23)	22 (26.51)
*1500~2000*	26 (35.14)	25 (32.05)	28 (38.36)	28 (34.15)	31 (39.74)	26 (31.33)
*2001~2500*	16 (21.62)	24 (30.77)	16 (21.92)	24 (29.27)	18 (23.08)	24 (28.92)
*2500~3000*	9 (12.16)	5 (6.41)	7 (9.59)	7 (8.54)	9 (11.54)	8 (9.64)
* > 3000*	6 (8.11)	3 (3.85)	6 (8.22)	3 (3.66)	5 (6.41)	3 (3.61)

Note: NCMS – New Cooperative Medical Scheme.

**Table 2:** Estimates of effect sizes derived from the difference-in-difference analyses.

	Intervention effect	95%CI	Z	*p*
**Prescriptions containing antibiotics**				
Bronchitis	0.02%	[−0.9%, 0.09%]	0.05	0.964
Gastritis	**−12.72%**	[−16.59%, −8.85%]	−6.45	< 0.001
Hypertension	**2.00%**	[0.53%, 3.47%]	2.67	0.008
**Prescriptions containing two or more antibiotics**				
Bronchitis	**−3.79%**	[−6.42%, −1.17%]	−2.83	0.005
Gastritis	−0.096%	[−1.56%, 1.37%]	−0.13	0.898
Hypertension	0.44%	[−0.041%, 0.91%]	1.79	0.073
**Prescriptions containing injections**				
Bronchitis	**2.00%**	[0.43%, 3.56%]	2.50	0.012
Gastritis	**−10.59%**	[−14.47%, -6.62%]	−5.22	< 0.001
Hypertension	−0.97%	[−3.37%, 1.43%]	−0.79	0.428
**Prescriptions containing antibiotic injections**				
Bronchitis	−0.075%	[−2.02%, 1.87%]	−0.08	0.939
Gastritis	**−10.73%**	[−14.41%, −7.04%]	−5.70	< 0.001
Hypertension	−0.18%	[−0.80%, 0.44%]	−0.57	0.569
**Average prescription cost (Chinese Yuan)**				
Bronchitis	**−7.90%**	[−10.22%, −5.59%]	−6.69	< 0.001
Gastritis	**−5.71%**	[−9.72%, −1.69%]	−2.79	0.005
Hypertension	−1.72%	[−5.61%, 2.18%]	−0.86	0.388

Note: CI - Confidence Interval.
