# Supplementary material for: Public reporting as a prescriptions quality improvement measure in primary care settings in China: variations in effects associated with diagnoses
Source: Sci Rep. 2016 Dec 20;6:39361. doi: 10.1038/srep39361 (PMC5172199; doi:10.1038/srep39361)
Supplement: Supplementary File [file srep39361-s1.doc]

**Public reporting as a prescriptions quality improvement measure in primary care settings in China: variations in effects associated with diagnoses**

**Yuqing Tang^1^, Chaojie Liu^2^, Xinping Zhang^1^***

**Table S1 Characteristics of participating institutions between groups**

| **Characteristics** | **Control group** | **Intervention group** |
| --- | --- | --- |
| Sample size | 10 | 10 |
| Population serviced (10,000) | 4.04 | 3.83 |
| Average number of beds | 65.60 | 60.00 |
| Average number of doctors | 28.30 | 26.30 |
| Average annual outpatient visits | 50199.60 | 49108.20 |
| Average annual episodes of admissions | 1348.60 | 1482.20 |
| Average annual revenue from drug sales (10,000 Yuan) | 188.87 | 150.78 |

**Segmented time series analysis**

**Methods**

We performed 10 segmented time series analyses for specific outcome indicators for specific groups using the methods recommended by Wagner and colleagues[^1^](#_ENREF_1):

Yt=β0+β1×time_t_+β2×intervention_t_ +β3×time after intervention_t_ +e (1)

In this model:

Y_t_ is the targeted outcome indicators (percentage of prescriptions containing antibiotics, two or more antibiotics, injections and antibiotic injections and average prescription cost) in month t;

time_t_ is a continuous variable indicating time in months at time t from the start of the observation period;

intervention_t_ receives a value of 0 before public reporting and 1 after it;

time after intervention_t_ receives a value of 0 before public reporting and is a continuous variable indicating time in months after public reporting;

β0 captures the baseline level prior to the interventions;

β1 captures the baseline time trend prior to the interventions;

β2 captures the level of changes after the interventions;

β3 captures the trend change after the time of intervention;

e was the error term.

For the indicators that showed a time trend, the following segmented equation[^2^](#_ENREF_2) was established, which allows us to separate the intervention effect.

Y_t_ = β0 + β1 ×time_t_ + β2 ×intervention_t_ + β3×time after interventiont_t_ + β4×group + β5×group ×time_t_ + β6×group× intervention_t_ + β7×group × time after intervention_t_ + e (2)

In this model:

group - 1 indicates “intervention group”; 0 indicates “control group”;

β4 estimates the mean difference of the outcome indicator between the control and intervention groups prior to the public reporting interventions;

β5 estimates the difference in time trend of the outcome indicator between the control and intervention groups prior to the pubic reporting interventions;

β6 estimates the mean difference in pre-post (interventions) changes of the outcome indicator between the control and intervention groups;

β7 estimates the difference in pre-post (interventions) time trend changes between the control and intervention groups.

Auto correlations were adjusted in all equations. Durbin-Watson statistic were calculated to detect the presence of autocorrelations.

**Results**

Baseline time trends were observed in the seven outcome indicators for both intervention and control groups: average prescription expenditure for bronchitis, gastritis and hypertension; combined use of antibiotics for bronchitis; prescriptions containing antibiotic injections for bronchitis; prescriptions containing injections for hypertension; and prescriptions containing antibiotic injections for hypertension (Table S2, Table S3 and Table S4).

Table S2 Prescription indicators for bronchitis - segmented time series regression

| **Variables** | **Intervention group** | **Control group** |
| --- | --- | --- |
| **Prescriptions containing antibiotics** | | |
| Baseline level | 93.24*** | 84.27*** |
| Baseline trend | -0.08 | -0.03 |
| Level change | 4.69** | -6.52 |
| Trend change | -0.33 | 0.93 |
| **Prescriptions containing two or more antibiotics** | | |
| Baseline level | 25.39*** | 18.86*** |
| Baseline trend | 1.79** | 2.38* |
| Level change | 14.21** | 19.95* |
| Trend change | -2.06** | -2.32* |
| **Prescriptions containing injections** | | |
| Baseline level | 84.94*** | 89.15*** |
| Baseline trend | -0.73 | -0.99 |
| Level change | -2.69 | -6.45 |
| Trend change | 0.64 | 1.05 |
| **Prescriptions containing antibiotic injections** | | |
| Baseline level | 82.86*** | 81.46*** |
| Baseline trend | -1.50* | -1.92* |
| Level change | -8.23 | -14.93* |
| Trend change | 1.59* | 2.53* |
| **Average expenditure per prescription (Chinese Yuan)** | | |
| Baseline level | 10.37*** | 4.47 |
| Baseline trend | 0.79 | 1.97* |
| Level change | 2.88 | 14.17 |
| Trend change | -0.32 | -1.69 |

Note: *p<0.05, **p<0.01,***p<0.001

Table S3 Prescription indicators for gastritis - segmented time series regression

| **Variables** | **Intervention group** | **Control group** |
| --- | --- | --- |
| **Prescriptions containing antibiotics** | | |
| Baseline level | 53.26*** | 36.99*** |
| Baseline trend | -0.26 | 2.23 |
| Level change | -1.09 | 1.29 |
| Trend change | -0.24 | -0.89 |
| **Prescriptions containing two or more antibiotics** | | |
| Baseline level | 6.02*** | 3.31* |
| Baseline trend | -0.18 | 0.44 |
| Level change | 2.99 | 3.34 |
| Trend change | -0.2 | -0.57 |
| **Prescriptions containing injections** | | |
| Baseline level | 47.44*** | 51.03*** |
| Baseline trend | 0.01 | -0.32 |
| Level change | -19.16* | -13.38 |
| Trend change | 0.93 | 1.46 |
| **Prescriptions containing antibiotic injections** | | |
| Baseline level | 25.96*** | 26.85*** |
| Baseline trend | 0.71 | 0.68 |
| Level change | -4 | -7.34 |
| Trend change | -0.41 | 0.83 |
| **Average expenditure per prescription (Chinese Yuan)** | | |
| Baseline level | 34.48*** | 30.20*** |
| Baseline trend | 0.63 | 2.08*** |
| Level change | 1.44 | 16.98*** |
| Trend change | -0.36 | -2.32*** |

Note: *p<0.05, **p<0.01,***p<0.001

Table S4 Prescription indicators for hypertension - segmented time series regression

| **Variables** | **Intervention group** | **Control group** |
| --- | --- | --- |
| **Prescriptions containing antibiotics** | | |
| Baseline level | 4.26*** | **6.60***** |
| Baseline trend | -0.06 | **0.04** |
| Level change | 0.34 | **-2.53** |
| Trend change | 0.16 | **0.05** |
| **Prescriptions containing two or more antibiotics** | | |
| Baseline level | 0.09 | 1.1 |
| Baseline trend | 0.06 | -0.04 |
| Level change | 0.7 | -1.49 |
| Trend change | -0.06 | 0.13 |
| **Prescriptions containing injections** | | |
| Baseline level | 12.97* | 42.71*** |
| Baseline trend | 0.32 | -3.50*** |
| Level change | 2.64 | -4.18 |
| Trend change | -0.28 | 2.19* |
| **Prescriptions containing antibiotic injections** | | |
| Baseline level | 2.28 | 7.59*** |
| Baseline trend | -0.15 | -1.09*** |
| Level change | -2.85 | -6.09*** |
| Trend change | 0.41 | 1.12*** |
| **Average expenditure per prescription (Chinese Yuan)** | | |
| Baseline level | 48.75*** | 39.38*** |
| Baseline trend | 0.54 | 2.26* |
| Level change | 13.74* | 5.91 |
| Trend change | -1.3 | -1.79 |

Note: *p<0.05, **p<0.01,***p<0.001

The controlled segmented time series analyses (equation 2) revealed the effects of public reporting interventions on those indicators with time trends (Table S5). The results are consistent with the findings from the DID analyses in relation to the following indicators: average expenditure per prescription for hypertension; prescriptions containing antibiotic injections for bronchitis and hypertension; and prescriptions containing injections for hypertension. No significant effects of public reporting interventions on the level and time trend of these indicators were found at p<0.05 level. However, the interventional effects on average prescription costs and combined use of antibiotics for bronchitis detected in the DID analyses were not found in the time trend analyses. The average prescription cost for gastritis decreased immediately (p<0.01) after the interventions, but the decrease was offset by a rising time trend after the intervention (p<0.01).

**Table S5 Effects of public reporting interventions - controlled segmented regression analyses**

| **variables** | **Level change** | **95%CI** | **Trend change** | **95%CI** |
| --- | --- | --- | --- | --- |
| **Average expenditure per prescription** | | | | |
| Bronchitis | -11.35 | [-27.86, 5.17] | 1.37 | [-0.87,3.62] |
| Gastritis | -16.23** | [-26.95,-5.51] | 2.09** | [0.62, 3.55] |
| Hypertension | 7.81 | [-10.53,26.16] | 0.5 | [-2.06,3.07] |
| **Prescriptions containing two or more antibiotics** | | | | |
| Bronchitis | -5.65 | [-22.51,11.22] | 0.32 | [-2.04,2.68] |
| **Prescriptions containing antibiotic injections** | | | | |
| Bronchitis | 6.80 | [-9.81,23.40] | -0.95 | [-3.20, 1.31] |
| Hypertension | 3.94 | [-0.96,8.84] | -0.75* | [-1.43,-0.07] |
| **Prescriptions containing injections** | | | | |
| Hypertension | 8.2 | [-12.85, 29.25] | -2.48 | [-5.38,0.41] |

Note: CI – Confidence Interval. Adjusted Durbin–Watson statistics in these models ranged between 1.65 and 2.08, indicating absence of auto correlations. *p<0.05, **p<0.01.

References

1. Wagner, A.K., Soumerai, S.B., Zhang, F. & Ross-Degnan, D. Segmented regression analysis of interrupted time series studies in medication use research. *J Clin Pharm Ther* **27**, 299-309 (2002).

2. Koskinen, H., Mikkola, H., Saastamoinen, L.K., Ahola, E. & Martikainen, J.E. Time Series Analysis on the Impact of Generic Substitution and Reference Pricing on Antipsychotic Costs in Finland. *Value in Health* **18**, 1105–1112 (2015).
